# Ribonomic analysis of human DZIP1 reveals its involvement in ribonucleoprotein complexes and stress granules

**DOI:** 10.1186/1471-2199-15-12

**Published:** 2014-07-03

**Authors:** Patrícia ShigunovShigunov, Jose Sotelo-Silveira, Marco Augusto Stimamiglio, Crisciele Kuligovski, Florencia Irigoín, Jose L Badano, David Munroe, Alejandro Correa, Bruno Dallagiovanna

**Affiliations:** 1 Stem Cells Basic Biology Laboratory, Instituto Carlos Chagas, FIOCRUZ, Algacyr Munhoz Mader 3775, Curitiba 81350-010, Brazil; 2 Genomics Department, Instituto de Investigaciones Biológicas Clemente Estable, Avenida Italia 3318, CP 11600 Montevideo, Uruguay; 3 Cell and Molecular Biology Department, Fac. Ciencias, Universidad de la República Uruguay, Avenida Italia 3318, CP 11600 Montevideo, Uruguay; 4 Institut Pasteur Montevideo, Mataojo 2020, Montevideo 11400, Uruguay; 5 Departamento de Histología y Embriología, Facultad de Medicina, Universidad de la República, Avenida General Flores 2125, CP 11800 Montevideo, Uruguay; 6 Cancer Research Technology Program, Leidos Biomedical Research, Inc., Frederick National Laboratory for Cancer Research, Frederick, MD 21702, USA

**Keywords:** DZIP1, Ribonucleoprotein, Stress granules, Polysome, Hedgehog signaling

## Abstract

**Background:**

DZIP1 (DAZ-interacting protein 1) has been described as a component of the Hh signaling pathway with a putative regulatory role in ciliogenesis. DZIP1 interacts with DAZ RNA binding proteins in embryonic stem cells and human germ cells suggesting a role in mRNA regulation.

**Results:**

We investigated DZIP1 function in HeLa cells and its involvement in ribonucleoprotein complexes. DZIP1 was predominantly located in granules in the cytoplasm. Under oxidative stress conditions, DZIP1 re-localized to stress granules. DZIP appears to be important for the formation of stress granules during the stress response. We used immunoprecipitation assays with antibodies against DZIP1 and microarray hybridization to identify mRNAs associated with DZIP1. The genetic networks formed by the DZIP1-associated mRNAs were involved in cell cycle and gene expression regulation. DZIP1 is involved in the Hedgehog signaling pathway. We used cyclopamine, a specific inhibitor of this pathway, to analyze the expression of DZIP1 and its associated mRNAs. The abundance of DZIP1-associated mRNAs increased with treatment; however, the silencing or overexpression of DZIP1 in HeLa cells had no effect on the accumulation of the associated mRNAs. Polysomal profile analysis by sucrose gradient centrifugation demonstrated the presence of DZIP1 in the polysomal fraction.

**Conclusions:**

Our results suggest that DZIP1 is part of an RNP complex that occupies various subcellular locations. The diversity of the mRNAs associated with DZIP1 suggests that this protein is a component of different RNPs associated with translating polysomes and with RNA granules.

## Background

The zebrafish *iguana* gene, also referred to as *DZIP1* (DAZ-interacting protein 1), has three protein isoforms, each with a single C2H2 zinc finger domain but no other defined domain [1]. The biological role of DZIP1 is still not clearly defined and it has been reported to be involved in the regulation of various molecular processes. The DZIP1 protein is a component of the Hedgehog (Hh) signaling pathway and has a putative regulatory role in Hh signaling and ciliogenesis [2-6]. The Hh signaling pathway is involved in many processes during embryonic development and remains active in adults, where it controls cell growth, survival and fate [7]. The principal mediators of the transcriptional response to Hh are members of the zinc finger-containing GLI protein family [8]. In vertebrates, Gli processing requires an intact primary cilium, which is a microtubule-based organelle on the cell surface. The integrity of the primary cilium is essential for mammalian Hh signaling [9]. DZIP1 regulates Gli turnover through stabilizing Speckle-type POZ protein (Spop) independent of its role in ciliogenesis [5,10]. DZIP1 is located at the basal body of the primary cilium [2,3]. Kim *et al.* (2010) suggested that DZIP1 may be an essential component of a protein complex involved in the biogenesis of the primary cilium.

Human DZIP1 associates with the RNA-binding protein DAZ in embryonic stem cells and germ cells, which led to the suggestion that it is involved in mRNA regulation [1]. The proteins of the DAZ family (DAZ, DAZ-like and BOULE) activate the translation of particular mRNAs in metazoan germ cells [11-14] by interacting with the poly(A)-binding protein (PABP) [12]. It has also been suggested that proteins of the DAZ family transport target transcripts to RNA granules [15]. Moreover, DAZL is an essential component of stress granules, which prevent male germ cells from undergoing apoptosis in conditions of heat stress [16]. Thus, DZIP1 may be a component of ribonucleoprotein (RNP) complexes.

We show here that DZIP1 is located predominantly in granules in the cytoplasm and that it is a component of ribonucleoprotein complexes in HeLa cells. We also found that DZIP1 is associated with polysomes and colocalizes with TIA-1 in stress granules, but not with p-bodies, suggesting a role in the localization of mRNAs within the body of the cell. Ribonomic analysis of associated mRNAs identified networks of genes involved principally in cell cycle regulation and gene expression. Our results suggest that DZIP1 is part of an RNA localization complex involved in regulating the cellular trafficking of a defined subpopulation of mRNAs.

## Results

### DZIP1 is present predominantly in the cytoplasm of HeLa cells

**Figure 1:**
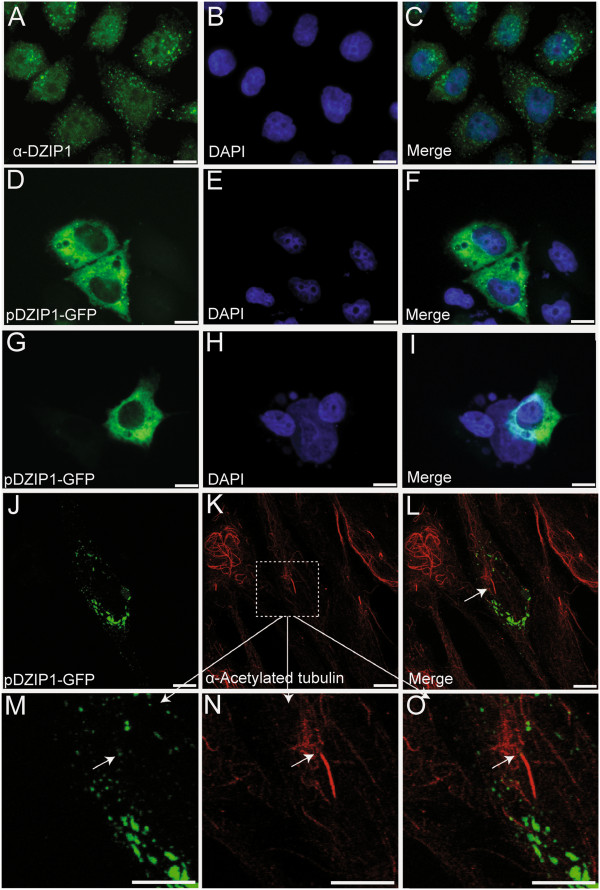
**DZIP1 is located predominantly in the cytoplasm and shows a granular distribution. (A)** Indirect immunofluorescence staining of DZIP1 (green) in HeLa cells. **(B)** Nuclei counterstained with DAPI (blue) and **(C)** merged image. HeLa **(D-F)**, HEK293 **(G-I)** and hTERT-RPE1 **(J-O)** cells were transfected with pDZIP1-GFP. **(J-O)** Ciliary axonemes were labeled with anti-acetylated tubulin antibodies (red). **(M-O)** are magnified sections from **(K)** (white boxes). Arrow = potentially compatible with a basal body localization. Scale bar: 10 μm.

We used indirect immunofluorescence with an anti-DZIP1 antibody and amino-GFP or carboxy-YFP-tagged hDZIP1 proteins to investigate the subcellular distribution of DZIP1 in HeLa cells. DZIP1 labeling showed a granular pattern in the cytoplasm, with a slightly stronger signal in the perinuclear region (Figure 1A-C). Furthermore, we observed some nuclear staining with the anti-DZIP1 antibody. We sought to confirm these results and transfected cells with a vector encoding a carboxy-GFP-tagged DZIP1 and observed after 24 hours. The tagged-DZIP1 protein was also present mostly in the cytoplasm (Figure 1D-F). We observed a similar pattern when HEK293 cells were transfected with this construct, although the granular distribution was less evident than in HeLa cells (Figure 1G-I). We also obtained similar results when we transfected cells with an amino-YFP-tagged DZIP1 construct (see Additional file 1: Figure S1I-L). We used the pGFP or pYFP plasmid as a control (see Additional file 1: Figure S1A-H).

Zebrafish Iguana proteins (DZIP1 and DZIP1L) are located at the basal body of primary cilia [3]. The human DZIP1 protein has also been reported to be present in the basal body of hTERT-RPE1 cells (epithelial cells immortalized with hTERT) [2]. We therefore investigated the distribution of GFP-tagged DZIP1 in hTERT-RPE1 cells, using an anti-acetylated tubulin antibody to visualize the primary cilium [17] (Figure 1J-O). Although the vast majority of DZIP1 signal is restricted to foci distributed throughout the cytoplasm (Figure 1J-O), we cannot discard that at least a small fraction of the protein might localize to the base of the cilium (Figure 1M-O, arrows). We obtained similar results with the YFP construct, but the signal was less intense (see Additional file 1: Figure S1M-O). The combination of diffuse and granular patterns of DZIP1 staining in the cytoplasm suggests that at least some of the protein is present in high molecular weight protein complexes. We used fluorescence recovery after photobleaching (FRAP) assays to determine the mobile/immobile fraction of GFP-tagged DZIP1. The mean mobile fraction was 0.64 (±0.19) and the immobile fraction was 0.36 (±0.19) (see Additional file 1: Figure S1P-S). The immobile fraction was larger than this mean value if photobleaching was carried out close to the nucleus (data not shown). These findings indicate that the DZIP1 protein is partitioned between soluble and insoluble forms.

### DZIP1 is recruited to stress granules in cells subjected to oxidative stress

**Figure 2:**
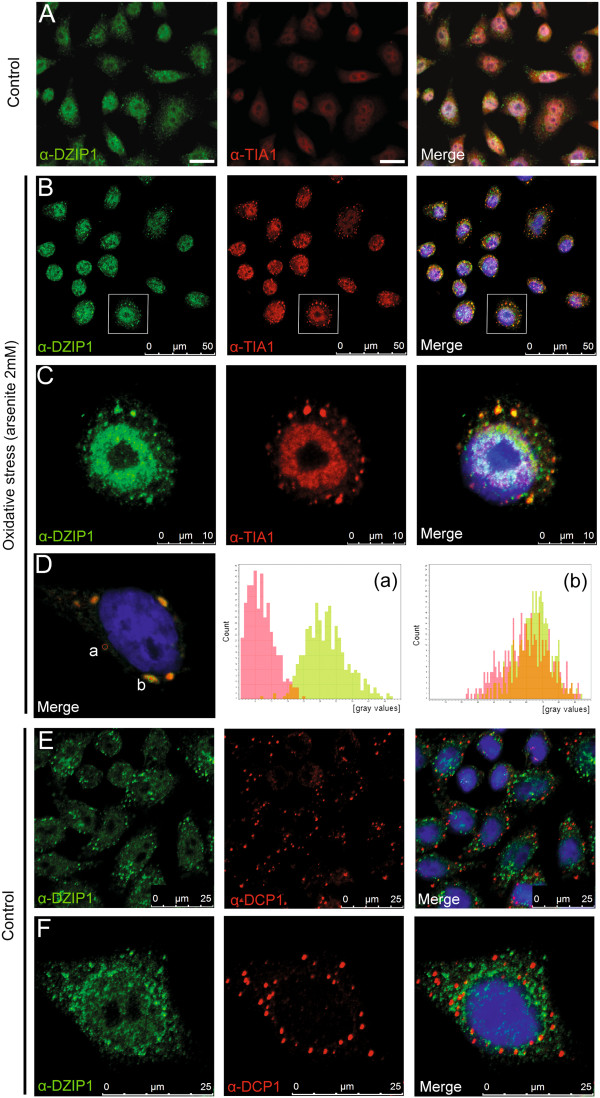
**DZIP1 is recruited to stress granules in cells under oxidative stress. (A-B)** Indirect immunofluorescence staining was carried out to examine the colocalization of DZIP1 (green) and TIA1 (red) in HeLa cells. Nuclei were counterstained with DAPI (blue). **(A)** Control cells without arsenite sodium (scale bar: 10 μm) and **(B)** cells treated with arsenite sodium to induce oxidative stress. **(C)** Images show magnified sections from **B** (white boxes). **(D)** Fluorescence intensities of DZIP1 (green channel) and TIA1 (red channel) were measured on selected regions (a and b) in the merged images (left) of HeLa cells under oxidative stress. The region (a) shows no colocalization of fluorescent signals with higher fluorescence intensity in the green channel than in the red channel. The region (b) shows DZIP1 and TIA1 colocalization with equivalent fluorescence intensities in green and red channels. **(E)** Indirect immunofluorescence staining of DZIP1 (green) and DCP1 (red) antibody was used to detect colocalization with p-bodies. **(F)** Detail of colocalization of DZIP1 and DCP1 in a single cell. **(B-F)** Confocal microscopy.

DAZL colocalizes with TIA1, a stress granule marker, in HeLa cells under oxidative stress conditions [18]. DZIP1 interacts with the DAZ and DAZL proteins in embryonic stem cells and germ cells [1]. Thus, DZIP1 may be a component of regulatory RNA granules. We used live cell imaging to observe DZIP1-GFP-expressing cells subjected to cold-shock treatment. Within a few minutes, DZIP1 was concentrated in cytoplasmic granules, the number and intensity of which increased over time (see Additional file 2: Figure S2A). We performed immunofluorescence assays with anti-DZIP1 and anti-TIA1 antibodies in transfected HeLa cells subjected to normal or oxidative stress conditions to investigate whether these granules corresponded to extensively studied stress granules (Figure 2A-C, see Additional file 2: Figure S2B). In normal conditions (control), DZIP1 colocalized with TIA-1 in the nucleus (Figure 2A). Arsenite treatment induced the formation of stress granules at concentrations of 0.5 mM - 2 mM, as demonstrated by labeling for TIA-1. DZIP1 mostly colocalized with TIA-1-containing granules (Figure 2B-C, see Additional file 2: Figure S2B). Interestingly, some DZIP1-containing granules were not stress granules, although the colocalization of DZIP1 with these granules was less robust than with stress granules. We verified that the TIA-1 signal was not leaking back into the FITC channel (Figure 2D). We investigated whether DZIP1 was present in other RNA granules, such as p-bodies, by labeling the cells for DCP1 which is a p-body marker. DZIP1 did not colocalize with this marker in HeLa cells either in normal conditions (Figure 2E-F) or under oxidative stress (see Additional file 2: Figure S2C). These results suggest that DZIP1 participates in ribonucleoprotein complexes under normal and stress conditions.

### DZIP1 is a component of ribonucleoprotein complexes and is associated with a particular subpopulation of mRNAs

**Figure 3:**
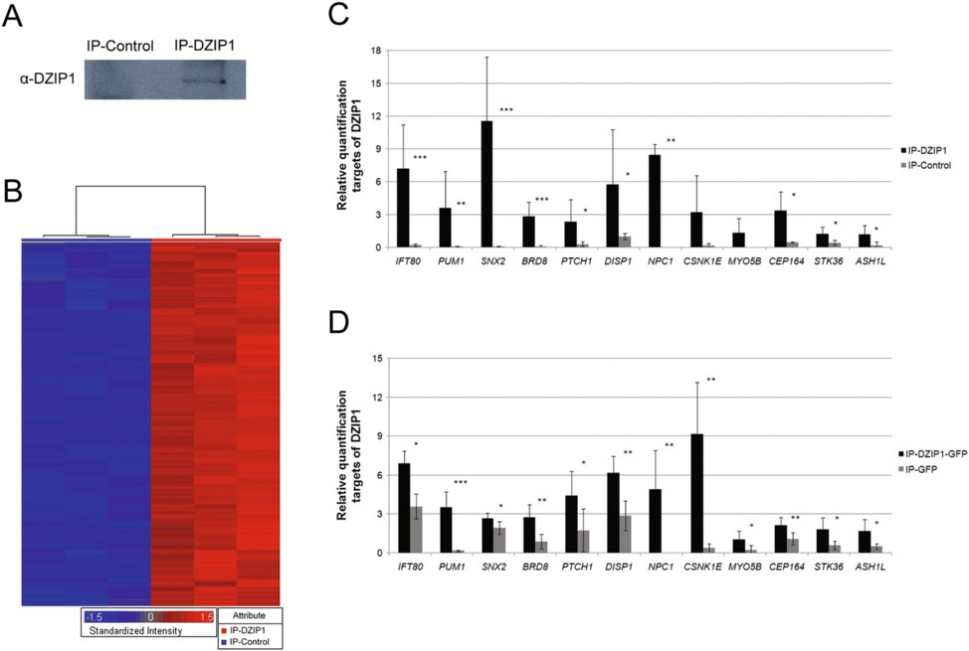
**Identification of DZIP1-associated mRNAs in HeLa cells. (A)** Western blot of HeLa cell proteins immunoprecipitated with a specific anti-DZIP1 antibody and a control IgG, probed with a specific anti-DZIP1 antibody; **(B)** mRNAs associated with DZIP1. Microarray analysis of the immunoprecipitated RNA fractions. Rows correspond to individual transcripts and the color code indicates the degree of enrichment. Three independent assays, with anti-DZIP1 antibody and negative controls, are shown. **(C)** Quantitative RT-PCR analysis of transcript levels in DZIP1-IPs (DZIP1 immunoprecipitate) versus negative IPs (IP-Control), normalized to concentration. Enrichment of the following mRNAs was analyzed: *IFT80, PUM1, SNX2, BRD8, PTCH*, *DISP1, NPC1, CSNK1E, MYO5B, CEP164, STK36* and *ASH1L*. The mean of technical triplicates is shown. **(D)** Quantitative RT-PCR of transcript levels in DZIP1-GFP IPs versus negative IPs (IP-GFP), normalized to concentration. Enrichment of the following mRNAs was analyzed: *IFT80, PUM1, SNX2, BRD8, PTCH*, *DISP1, NPC1, CSNK1E, MYO5B, CEP164, STK36* and *ASH1L.* The mean of technical triplicates is shown.

DZIP protein interacts with DAZ (deleted in azoospermia) in embryonic stem cells and germ cells [1]. The genes of the *DAZ* family encode RNA-binding proteins [15]. We therefore carried out immunoprecipitation assays with anti-DZIP1 antibodies to determine whether DZIP1 was present in ribonucleoprotein complexes in HeLa cells in normal conditions and to identify the mRNAs with which it was associated. We confirmed the presence of DZIP1 in the immunoprecipitates by western blotting (Figure 3A) and we identified the mRNAs present in the immunoprecipitates by microarray hybridization, with a GeneChip Affymetrix Human Genome U133 Plus 2.0 Array (Figure 3B). As a control, we used a rabbit isotype IgG antibody (IP-Control). All signals that were two-fold higher than the control were considered as positive. In total, 585 genes displayed four-fold enrichment, and 1737 displayed two-fold enrichment in the DZIP1 elutes (see Additional file 3: Table S1). We tested twelve microarray-positive candidates by quantitative RT-PCR to validate our microarray results (Figure 3C). We chose to analyze genes associated with the Hedgehog signaling pathway (PTCH1, CSNK1E, STK36, DISP1 and NPC1), genes associated with cilia or intraflagellar transport (CEP164 and IFT80), motor proteins and their associated proteins (MYO5B and ASH1L) and randomly selected genes with different levels of fold change (SNX2 – fold change 82, BRD8 – fold change 20 and PUM1 – fold change 7.6). Ten of them showed statistically significant enrichment in the DZIP1-IP fractions.

We also transfected Hela cells with a plasmid encoding GFP-tagged DZIP1 (see Additional file 4: Figure S3A) and carried out immunoprecipitation assays with an anti-GFP antibody. We used cells transfected with pGFP alone as a negative control. We reverse transcribed the mRNAs associated with the immunoprecipitated GFP-tagged DZIP1 and analyzed the same 12 microarray-positive candidate genes by quantitative PCR all of them being enriched in this fraction (Figure 3D).

We analyzed the functional relationships between DZIP1-associated mRNAs by assigning biological functions on the basis of Gene Ontology terms, and we used IPA to identify the gene networks formed by the transcripts. GO analysis showed enrichment in regulatory proteins. IPA identified gene networks involved in the control of cell growth, gene expression and cellular compromise (Table 1).

**Table 1:** Molecular and cellular functions associated with DZIP1 targets

**Category**^**a**^	**Top-ranked functions**	**Enrichment score**
Binding function	Protein binding	28.69
	Nucleic acid binding	21.78
	Chromatin binding	4.37
Molecular function	Binding	23.48
	Catalytic activity	7.19
	Transcription regulator activity	3.2
	Molecular transducer activity	2.45
	Structural molecule activity	2.08
Components	Extracellular region	16.22
	Cell part	11.21
**Associated network functions**^**b**^	***p-*** **value**	**No. of molecules**
Cell cycle	3.07E-12 - 4.89E-02	127
Gene expression	1.22E-10 - 4.65E-02	153
Cellular compromise	1.35E-10 - 2.95E-02	107
DNA replication, recombination, and repair	1.06E-09 - 3.61E-02	113
RNA posttranscriptional modification	7.52E-09 - 1.65E-02	44

^a^GO annotations of DZIP1 mRNA targets.

^b^Ingenuity pathway analysis networks: Functions associated with the networks regulated by DZIP1.

DZIP1 contains a single C2H2 zinc finger domain. Zinc finger proteins are generally thought of as DNA-binding transcription factors. However, some classes of zinc finger proteins, including the common C2H2 zinc fingers, function as RNA-binding proteins [19]. We used EMSA to investigate the ability of DZIP1 to interact directly with RNAs. We produced a Myc-tagged protein in 293T cells and purified this protein by affinity chromatography (see Additional file 4: Figure S3B). We subsequently used this recombinant protein in EMSA with four homoribopolymer probes. Under the conditions used, DZIP1 did not interact robustly with the RNA probes, even in the most permissive condition tested (see Additional file 4: Figure S3C). These results suggest that DZIP1 is associated with an RNA binding protein which is associated directly with the mRNA population.

### The expression of DZIP1 and its mRNAs targets is affected by inhibition of the Hh pathway

**Figure 4:**
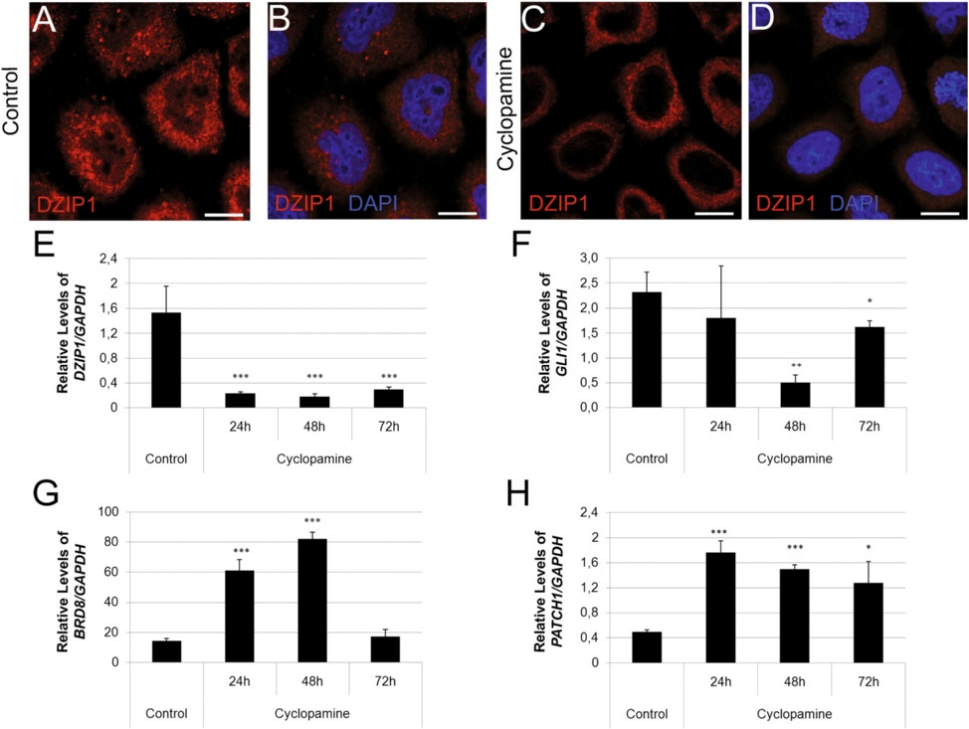
**The expression of DZIP1 and its mRNA targets is affected by blockade of the Hh pathway. (A-D)** Indirect immunofluorescence staining was used to detect DZIP1 (red – alexa546), and nuclei were counterstained with DAPI (blue). **(A-B)** HeLa cells not treated with cyclopamine. **(C-D)** HeLa cells treated with 300 nM cyclopamine for 24 h. **(E-H)** Quantitative RT-PCR analysis of *DZIP1, GLI1, BRD8* and *PTCH1* mRNA levels in HeLa cells treated with cyclopamine for 24, 48 or 72 h. *GAPDH* was used as an internal housekeeping gene control. *P ≤ 0.05, **P ≤ 0.01, ***P ≤ 0.001. Scale bar: 10 μm.

DZIP1 has been described as a component of the Hh signaling cascade. Cyclopamine is a specific Hh inhibitor that blocks the activation of the Hh pathway by binding directly to Smo [20]. We first incubated HeLa cells with various concentrations of cyclopamine and determined the shortest time and the minimum concentration required to inhibit cell proliferation (see Additional file 5: Figure S4A). We evaluated proliferation by the incorporation of BrdU. The rate of cell proliferation was lower in cells incubated for 24 hours with 300 nM or 600 nM cyclopamine than in control cells. We assessed the abundance of Gli1 mRNA, a positive effector of Hh signaling, to evaluate the molecular effect of cyclopamine treatment (see Additional file 5: Figure S4B) [8]. The abundance of Gli1 mRNA was considerably lower in cells incubated with 300 nM cyclopamine than in control cells. We examined cell cycle progression and apoptosis to determine the optimal concentration of cyclosporine to block the Hh pathway without modifying the cell cycle or viability. Cell cycle progression and the percentage of apoptotic cells were similar in cells treated with 300 nM cyclopamine for 24 hours and in untreated control cells (see Additional file 5: Figure S4C-F). We therefore incubated cells with 300 nM cyclopamine for 24 hours in subsequent experiments.We investigated the effect of blocking the Hh pathway on the subcellular distribution of DZIP1. We treated HeLa cells with cyclopamine and carried out immunolocalization assays with an anti-DZIP1 antibody. Fewer granules were present in the cytoplasm in cells in which the Hh pathway was blocked with cyclopamine than in untreated control cells (Figure 4A-D). Moreover, the nuclear signal was completely absent after treatment. The low number of granules and absence of nuclear signal may be connected with inhibition of the Hh signaling pathway.

We investigated the abundance of *DZIP1* and *GLI1* transcripts and that of two DZIP1-associated mRNAs (*BRD8* and *PTCH1*) after treatment with cyclopamine for 24, 48 or 72 hours. Blockade of the Hh pathway prevented the accumulation of the *DZIP1* and *GLI1* transcripts (Figure 4E-F). This treatment also promoted the accumulation of *PTCH1* and *BRD8* transcripts (Figure 4G-H). The high abundance of *PTCH1* and *BRD8* mRNA may be a direct effect of blocking the Hh pathway or may be due to an impairment in *DZIP1* expression.

### Knockdown of DZIP1 expression affects cell proliferation

**Figure 5:**
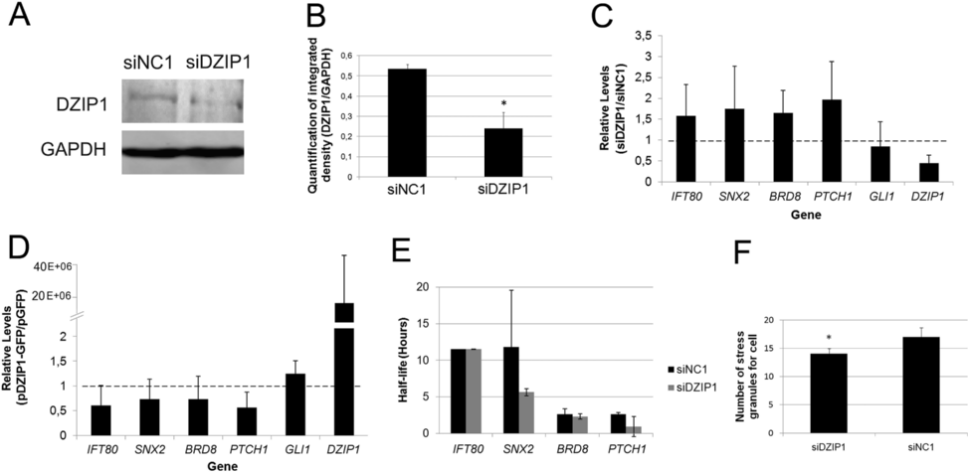
***DZIP1 *knockdown and overexpression do not affect the accumulation or stability of mRNAs associated with DZIP1-containing complexes but modify the quantity of stress granules per cell. (A)** Protein extracts from *DZIP1* knockdown (siDZIP1) and negative control (siNC1) cells after 72 h of transfection were analyzed by western blotting with antibodies against DZIP1 and GAPDH. **(B)** Western blots shown in **(A)** were used for quantitation and are representative of results from two experiments. **(C)** Quantitative RT-PCR analysis of *DZIP1, GLI1, PTCH1, BRD8*, *SNX2* and *IFT80* mRNA levels in *DZIP1* knockdown cells. **(D)** Quantitative RT-PCR analysis of *DZIP1, GLI1, PTCH1, BRD8, SNX2* and *IFT80* mRNA levels in HeLa cells transfected with pDZIP1-GFP or pGFP alone. *GAPDH* was used as an internal housekeeping gene control. **(E)** Mean half-life for some of the mRNA targets of DZIP1 (*IFT80, SNX2, BRD8* and *PTCH1*) in HeLa cells transfected with siDZIP1 or siNC1. Triplicates were performed for qRT-PCR and the mean half-life was obtained from two independent experiments. **(F)** DZIP1 knockdown cells and control (NC1) were subjected to oxidative stress and the formation of stress granules was determined by TIA1 staining. The granules were counted and statistically analyzed. At least 5 fields per slide (technical triplicate) were counted. *P ≤ 0.05.

We used DZIP1-specific siRNA molecules to investigate the role of DZIP1 and its effect on its associated mRNAs and determined whether the silencing of DZIP1 resulted in phenotypic changes or altered the abundance of associated mRNAs. We transfected HeLa cells with a mixture of DZIP1 siRNAs at a concentration of 1 nM and examined *DZIP1* expression at 24, 48, and 72 h after transfection (see Additional file 6: Figure S5A). Western blotting showed that the abundance of DZIP1 in protein extracts from transfected cells at 72 h was half that in cells transfected with a scrambled control (siNC1 – negative control), thus confirming the knockdown of *DZIP1* expression (Figure 5A-B). *DZIP1* knockdown had no significant effect on cell morphology. We carried out Annexin V assays to evaluate the survival rate of *DZIP1-*knockdown cells 72 hours after transfection by determining the percentage of living, apoptotic and dead cells. We found no significant differences between *DZIP1*-knockdown cells and control cells (see Additional file 6: Figure S5B-C). Propidium iodide staining also showed no differences in the percentages of the cells in the G1, S and G2 phases of the cell cycle (see Additional file 6: Figure S5D-E).

Kikuyama *et al*. (2012) recently described DZIP1 as a putative tumor suppressor gene. Indeed, *DZIP1-*knockdown in breast cancer cell lines promotes cell growth. We assessed the putative role for DZIP1 in the control of cell proliferation by evaluating the effect of *DZIP1* knockdown on the proliferation of HeLa cells. Growth curves showed that the knockdown population contained a higher number of dividing cells than the control population, as previously reported for tumor cells (see Additional file 6: Figure S5F).

### *DZIP1* knockdown and overexpression do not affect the accumulation or stability of mRNAs associated with DZIP1-containing complexes but modify the quantity of stress granules per cell

RNP complexes control the fate of bound mRNAs by regulating their stability, translation or subcellular localization [21]. We evaluated the abundance of *IFT80, SNX2, BRD8* and *PATCH1* mRNA in *DZIP1*-knockdown cells. We used *GLI1* mRNA as a non-target control. There was no statistical difference in the abundance of any of these mRNA (*IFT80, SNX2, PATCH1*, *BRD8* and *GLI1*) between knockdown and control cells, although knockdown cells tended to have higher levels of these transcripts than control cells (Figure 5C).

We next examined the relationship between the amount of DZIP1 and the accumulation of associated mRNAs in DZIP1-containing complexes in cells overexpressing DZIP1-GFP. We found that the abundance of the DZIP1-associated mRNAs *IFT80, SNX2, BRD8,* and *PTCH1* and the control mRNA *GLI1* were unaffected by the strong overexpression of *DZIP1* (Figure 5D).

We sought to investigate the potential role of DZIP1 in the regulation of the stability of the mRNA targets of RNP complexes; therefore, we used quantitative RT-PCR to determine the half-lives of mRNAs associated with DZIP1 in cells treated with actinomycin D. We transfected cells with siDZIP1 or siNC1 and treated them with the transcriptional inhibitor (10 μg/ml actinomycin D) for 0, 1, 2 or 4 hours at 72 hours after transfection. We used quantitative RT-PCR to determine the mean relative mRNA levels at each time point after the addition of Act D, and plotted these values to calculate the half-life of *BRD8, IFT80, SNX2* and *PTCH1* mRNA. There was no significant difference in the half-lives of these four DZIP1-associated mRNAs between DZIP1-knockdown cells and control cells (Figure 5E, see Additional file 7: Figure S6). We counted the number of stress granules per cell after 72 hours of transfection in *DZIP1*-knockdown cells grown in oxidative stress conditions, using TIA1 as a stress granule marker. The number of stress granules per cell was significantly lower in the *DZIP1*-knockdown cells than in the control (siNC1) cells (Figure 5F, see Additional file 6: Figure S5G). These results indicate that DZIP1 participates in the formation of stress granules upon oxidative stress.

### DZIP1 is associated with polysomes

**Figure 6:**
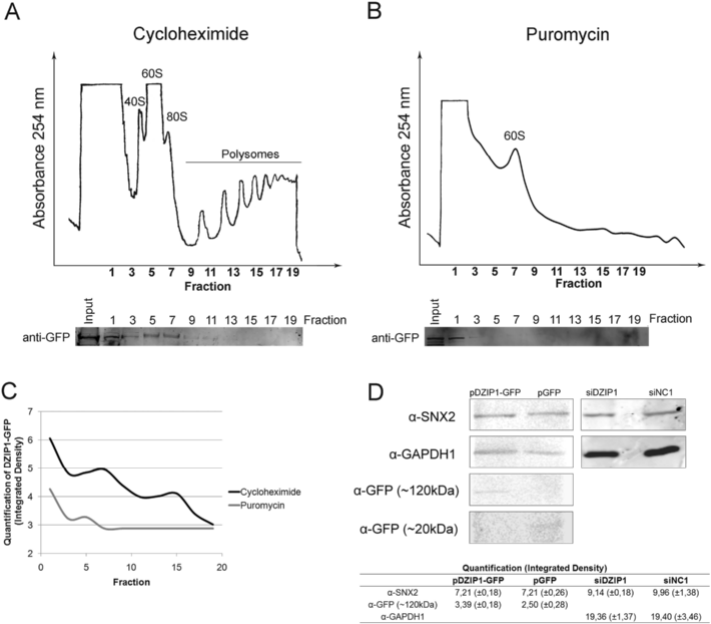
**Polysomal distribution of DZIP1-GFP. (A)** Lysates were prepared from HeLa cells treated with cycloheximide and were separated in a 10–50% sucrose gradient. Distribution of DZIP1-GFP was examined by western blotting. **(B)** HeLa cells were treated with puromycin, and extracts were prepared and processed as in **(A)**. **(C)** Quantitation of the distribution of DZIP1-GFP after cycloheximide and puromycin treatment. Western blots shown in **(A)** and **(B)** were used for quantitation. **(D)** Protein extracts of Hela cells transfected with pDZIP1-GFP, pGFP alone (control sample), siDZIP1 or siNC1 were analyzed by western blotting with antibodies against SNX2, GAPDH1 and GFP. Quantitation by integrated density of SNX2, DZIP1-GFP (α-GFP ~120 kDa) or GAPDH1 is shown in the table above.

**Figure 7:**
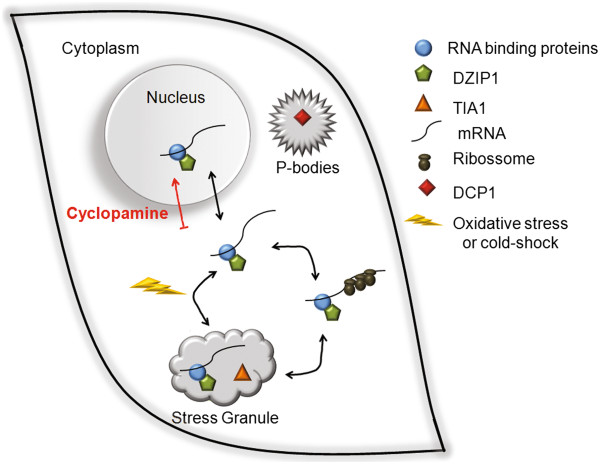
**Model of ribonucleoprotein complexes containing DZIP1 in stress granules.** DZIP1 is located mainly in the cytoplasm, where it is involved in ribonucleoprotein complexes related to mRNA networks involved primarily in controlling cell cycle and gene expression in HeLa cells. Inhibiting the Hedgehog pathway with cyclopamine blocks its translocation to the nucleus. DZIP1 is associated with polysomes and when HeLa cells are exposed to oxidative stress, DZIP1 is translocated to stress granules but not to processing bodies (p-bodies).

We evaluated the presence of DZIP1 in the polysomal fraction to analyze the association of DZIP1 with macromolecules involved in translation. We treated cells transfected with pDZIP1-GFP with cycloheximide to stabilize ribosome-mRNA complexes or with puromycin to disassemble ribosomes, and we separated cytoplasmic extracts by sucrose gradient sedimentation to isolate distinct ribosome populations (Figure 6A-B). We carried out western blotting to examine the distribution of DZIP1-GFP in both conditions using an antibody against GFP. Western blots shown in Figure 6A and B were used for quantification (Figure 6C). DZIP1-GFP was present in fractions 1–13 comprising 40S, 60S, 80S or monosomes and in polysome fractions. When cells were treated with puromycin, GFP-DZIP1 was only detected in the ribosome-free fractions (fractions 1–3), demonstrating that DZIP1-GFP is associated with translationally active ribosomes.DZIP1 protein complexes may regulate the translation of the associated mRNAs. We tested this hypothesis by carrying out western blots with extracts of cells transfected with GFP-tagged pDZIP1, pGFP, siDZIP1 or siNC1, using anti-SNX2, anti-GFP and/or anti-GAPDH antibodies. The amount of SNX2 protein was similar in all conditions (Figure 6D). Although we analyzed only one target of DZIP, these results nonetheless indicate that DZIP1 does not influence the abundance proteins encoded by the associated mRNAs.Our results suggest that DZIP1 is a component of ribonucleoprotein complexes. DZIP1 does not regulate the expression of its associated mRNAs but is probably responsible for the subcellular localization of these transcripts to various types of ribonucleoprotein complexes (Figure 7). Thus, DZIP1 may be involved in the trafficking of its target mRNAs within the cell, i.e. between the nucleus, cytoplasm, stress granules and polysomes.

## Discussion

In this study, we found that DZIP1 was present mostly in the cytoplasm of HeLa cells and that this subcellular distribution was modified by external stimuli. Ribonomic analysis showed that DZIP1 was present in RNP complexes and was associated with a population of mRNAs involved principally in cell cycle regulation and the response to Hedgehog signaling.

Human DZIP1 and DZIPL are required for the formation of primary cilia [2]. DZIP1 is located at the basal body of cilia and its knockdown impairs ciliogenesis [3]. Cilia are necessary for Hh signaling in vertebrates [9]. Several Hedgehog signaling components are located at cilia in vertebrates, including the Patched and Smoothened transmembrane proteins [22] and Gli transcription factors [23]. These observations led to the suggestion that DZIP1 is involved in regulating the biogenesis of primary cilia and that its role in Hh signaling is related to its specific location in this cellular structure. Our data do not exclude the possibility that DZIP1 is present in the basal body of primary cilia, but we found that DZIP1 was present throughout the cytoplasm and, to a certain extent, in the nuclear compartment. Moreover, half of the protein appeared to be immobilized in granular structures, suggesting that this protein is a component of macromolecular complexes. This observation is consistent with the original report by Moore *et al*. (2004), who also observed DZIP1 in the cytoplasm of germ cells and reported that its translocation to the nucleus was dependent on its PKA-regulated phosphorylation [24].

*In situ* hybridization has shown that the abundance of Patched is low in zebrafish DZIP1 mutants [6]. Here, qRT-PCR revealed that DZIP1 knockdown did not affect Patched mRNA levels in HeLa cells. This can be explained by the different technique used to reduce the expression of DZIP1 (knockout vs. knockdown), because some residual expression will be present after gene knockdown. Alternatively, this discrepancy may be due to the different techniques used for the detection of mRNAs. Wang *et al.* (2013) showed that Cep164 protein fails to localize to ciliary appendages in DZIP1 mutant cells. Interestingly, we found that Cep164 mRNA (fold change +4.3) is regulated by a complex containing DZIP1 (Additional file 3: Table S1). DZIP1 regulates both ciliogenesis and the sequestration of Gli3 in the cytoplasm. These dual functions appear to be independent of each other and unique to DZIP1 [6]. Here we propose a third function of DZIP1 and show that DZIP1 is present in protein complexes involved in the regulation of mRNAs. Many aspects of the RNA regulon model [25] are reflected in the results presented here. Posttranscriptional regulation involves multifunctional proteins, which form ribonucleoprotein complexes that are assembled in a combinatorial manner. A single protein or mRNA may, therefore, be involved in several RNA regulons. Our ribonomic analysis showed that DZIP1 was associated with a vast subpopulation of mRNAs. These transcripts mostly encoded proteins involved in the control of the cell cycle and gene expression. DZIP1 has recently been identified as a putative tumor suppressor involved in controlling cell proliferation [26]. DZIP1 is also involved in the regulation of Hedgehog signaling, a pathway activated in several types of human cancers [27]. Thus, the stimulation of cell growth that we observed following *DZIP1* knockdown may reflect the derepression of the mRNA targets of DZIP1-containing regulatory RNP complexes. Interestingly, we found that several transcripts involved in the Hh response and in the biogenesis of primary cilia were associated with DZIP1. Various authors have suggested that DZIP1 plays an essential role in regulating ciliogenesis and is a structural component of the primary cilium [2,3]. We demonstrated that the activation state of the Hh signaling pathway determines the abundance of DZIP1 and its cellular distribution. Thus, DZIP1 may be indirectly involved in the formation of cilia and in Hh signaling, by influencing the fate of transcripts encoding various components of the two pathways.

The stability and translation of the identified transcripts also seemed to be unaffected by changes in the abundance of DZIP1. However, DZIP1 was clearly associated with polysomes. RNP complexes can also regulate the fate of target mRNAs by determining their distribution in specific foci in the cytoplasm, enhancing their translation or controlling their degradation [28]. The granular pattern of DZIP1 labeling was abolished by blockade of the Hh pathway. Moreover, DZIP1 was mobilized to RNA stress granules in response to heat or oxidative stress. DAZ family proteins (which interact with DZIP1) have also been reported to be associated with RNA granules during the stress response [16]. Knockdown of DZIP1 reduced the number of stress granules after oxidative stress. This suggests that DZIP1 is needed for the assembly of these RNA granules though it may be not a core component of them.

According to the RNA regulon model, the protein and mRNA contents of RNPs are dynamic, and vary in response to changing cellular conditions. It would therefore be of interest to determine the protein composition of these RNPs and to investigate how the stability, distribution and translation of DZIP1-target mRNAs change in response to cell stress. In addition, it is still unclear whether DZIP1 is essential for the formation of stress granules and the proteins that interact with DZIP1 in various conditions (normal, oxidative stress, blockade or activation of Hh signaling) remain to be determined.

## Conclusions

Our results suggest that DZIP1 is part of an RNP complex that occupies various subcellular locations. We propose a model in which DZIP1 interacts with various RNA-binding proteins, and moves from the nucleus through the cytoplasm where it binds to polysomes or storage/degradation complexes. DZIP appears to be important for the formation of stress granules during the stress response. The diversity of the mRNAs associated with DZIP1 suggests that this protein is a component of different RNPs associated with translating polysomes and with RNA granules.

## Methods

### Plasmids

All DZIP1 constructs were derived from human DZIP1 Ultimate™ ORF Clone -IOH27736 (Invitrogen). Bacterial expression constructs and DZIP1 mammalian expression plasmids (pEGFP, pEYFP and pSECTAG2B – Invitrogen) were constructed by PCR amplification and standard cloning methods. Cloning details are available on request.

### Cell culture, transfection and treatments

HeLa cells were maintained in RPMI-1640 medium supplemented with 10% heat-inactivated FCS, 100 U/ml penicillin and 100 U/ml streptomycin, at 37°C under a humidified atmosphere containing 5% CO_2_. The Nucleofector® kit, program I-013, was used to transfect 10^6^ cells with 5 μg plasmid DNA in 100 μl Opti-MEM® (Invitrogen). The Hh pathway was inhibited by incubating cultures with 300 nM cyclopamine for 24 h, after which RNA was extracted from the cells. Oxidative stress was induced by incubating the cells with 0.5 or 2.0 mM sodium arsenite (Sigma-Aldrich) for 10 minutes. Cold shock was performed by incubating the cells at 20°C and monitored for 30 minutes.

### Measurement of mRNA half-life

We assessed mRNA stability by adding actinomycin D (Sigma, cat #A-9415) to the medium at a concentration of 10 mg/ml to block transcription. Cells were harvested 0, 1, 2 and 4 h after the addition of actinomycin D. Total RNA was isolated and its concentration determined. First strand cDNA was synthesized from 1 μg of total RNA (DNase-treated). The mean relative amount of cDNA determined by quantitative RT-PCR at each time point after the addition of Act D was used to estimate the half-life of the transcript (t1/2) from a first-order decay model based on the equation, γ = β_0_ e^β^_1_^t^ + ϵ, where γ is the mean relative amount of mRNA at time t after the addition of Act D, β_0_ is the initial amount, β_1_ is a decay parameter related to half-life (t1/2 = ±ln2/ β_1_) and e is an error term [29,30].

### Immunofluorescence and FRAP (fluorescence recovery after photobleaching)

Cells seeded on glass coverslips were fixed in 4% formaldehyde solution for 10 minutes and washed with PBS. They were then permeabilized with 0.5% Triton X-100 in PBS for 30 min. Nonspecific binding sites were blocked with 5% BSA for 1 h, and the cells were then incubated for 1 h at 37°C with primary antibodies diluted in PBS containing 1% BSA at the following dilutions: anti-DZIP1 antibody (rabbit polyclonal, Santa Cruz Biotechnology) 1:30, anti-acetylated tubulin antibody (mouse monoclonal, Sigma) 1:1000, anti-DCP1a antibody (mouse monoclonal, Santa Cruz Biotechnology) 1:30, and anti-TIA1 antibody (goat polyclonal Santa Cruz Biotechnology) 1:50. The cells were incubated for 1 h at 37°C with secondary antibodies used at the following dilutions: anti-rabbit Alexa Fluor 488 (donkey) or anti-rabbit Alexa Fluor 546 (goat) both 1:500, anti-mouse rhodamine (goat) 1:1000, anti-mouse Alexa Fluor 594 (goat) both 1:500, or anti-goat Alexa Fluor 546 (donkey) 1:500. Cell nuclei were stained with DAPI. Images were obtained with a Nikon E-600 microscope or with a Leica SP5 laser scanning confocal microscope. The fluorescence intensity of green and red channels was used to qualify colocalization. The fluorescence intensity in each channel on selected regions of merged images was displayed as a histogram. Equivalent intensities in green and red channels correspond to signal colocalization.

For the assessment of the number of stress granules, DZIP1 knockdown cells and control (NC1) cells were subjected to oxidative stress and the formation of stress granules was determined by the localization of TIA1. The granules were counted manually with the aid of ImageJ software and analyzed statistically. At least five randomly selected fields per coverslips (technical triplicates) were counted. FRAP experiments were performed on a Leica SP5 confocal microscope with a 100×/1.3 NA oil-immersion objective and a 40 mW argon laser. Cells were imaged in LabTek II chambers (Nalgene) *in vivo*. Recovery data were binned logarithmically, generating a relatively uniform spacing of points along the FRAP curve, so as not to bias one phase of the curve when fitting the FRAP model [31].

### Extraction of total RNA and quantitative reverse transcription-polymerase chain reaction

RNA was extracted with the RNeasy mini kit (Qiagen). RT-PCR and quantitative RT-PCR were performed as described previously [32]. For a list of the primers used, see supporting Additional file 8: Table S2. GAPDH was used as an internal control. Experiments were performed with technical triplicates. Student’s *t* test was used to assess the significance of differences between the cell populations analyzed. *P*-values ≤ 0.05 were considered to be statistically significant.

### Western blotting

Protein extracts were obtained as described previously [33]. The rabbit anti-hDZIP1 antibody (1:200; Santa Cruz Biotechnology, CA, USA), rabbit anti-GFP (1:1000; Novus Biologicals), rabbit anti-GAPDH (1:200; Cell Signaling Technology) and mouse anti-SNX2 (1:1000; BD Bioscience) antibodies were used. Western blots were probed with anti-mouse and anti-rabbit dy680 secondary antibodies, and were then scanned and analyzed with the Odyssey infrared imaging system (Li-Cor Biosciences, Bad Homburg, Germany).

### Immunoprecipitation

Immunoprecipitation (IP) reactions were performed as described by Shigunov *et al.* (2012). For IP assays, we used 2 μg of anti-DZIP1 antibody (rabbit polyclonal, Santa Cruz Biotechnology, CA, USA) or 2 μg of anti-GFP antibody (NB600-308, rabbit polyclonal, Novus Biologicals, CO, USA) bound to protein A-agarose beads (Sigma, Deisenhofen, Germany) in three independent assays. HeLa cells were lysed in polysome lysis buffer (15 mM Tris–HCl pH 7.4, 15 mM MgCl_2_, 0.3 M NaCl, 1% Triton X-100, 1 mM DTT, 100 U/ml RNase Out, 1 mM PMSF and 10 μM E64) for 1 h at 4°C. The beads were washed, buffer and cell lysate were added, and the reaction mixtures were rotated vertically for 2 h at 4°C. The beads were then thoroughly washed again with polysome lysis buffer and then either boiled in denaturing buffer for western blots or used for RNA extraction for microarray and quantitative RT-PCR experiments. Identical IP experiments were performed with beads precoated with rabbit IgG as a negative control or with anti-GFP antibody for HeLa cells transfected only with pGFP.

### Microarray analysis

RNA was processed for hybridization with GeneChip 3′ IVT Express (Affymetrix - Santa Clara, USA), in accordance with the manufacturer’s instructions. Briefly, cDNA was synthesized from immunoprecipitated RNA by reverse transcription, followed by second-strand synthesis to generate double-stranded cDNA. An *in vitro* transcription reaction was used to generate biotinylated cRNA. The cRNA was purified and fragmented, and then hybridized onto GeneChip Affymetrix Human Genome U133 Plus 2.0 arrays. Post hybridization washes were performed with an Affymetrix GeneChip® Fluidics Station 450. Arrays were scanned on an Affymetrix GeneChip® Scanner 3000. Scanned arrays were normalized with the GCRMA in Partek software (Partek Incorporated. St. Louis, MO). Signal intensity ratios were calculated and a list of genes displaying a fold-change in abundance (IP-DZIP1/IP-negative) of at least 2.0 was generated. The list obtained was used as an input for Ingenuity pathway analysis (IPA), to determine the functional relationships between the mRNA enriched in DZIP1 immunoprecipitations. All microarray data were submitted to the GEO database and can be found under accession number GSE28882.

### RNA interference assays

The chemically synthesized dsRNA sequences for the Dicer-substrate 27-mers used in this study were synthesized and purified by HPLC (Integrated DNA Technologies, Coralville, IA). The non silencing dsRNA controls (NC1) included 27mers (Integrated DNA Technologies). Transfections were performed in 6-well plates, with Lipofectamine™2000 reagent (Invitrogen) used to deliver dsRNA into HeLa cells, in accordance with the manufacturer’s protocol. The final concentration of three DZIP1-specific dicer-substrate dsRNA mixtures and of the NC1 dsRNA was 1 nM and the lipid concentration was 5 μl/ml of medium. We determined the abundance of mRNA 24–72 h after transfection.

### Sucrose gradient analysis

Cells were then treated with 100 μg/ml cycloheximide for 10 min at 37°C followed by two washes on ice with cold PBS containing 100 μg/ml cycloheximide. Lysates were prepared, and gradient separation and fractionation were performed as described previously [34]. Cell lysis was performed for 10 minutes on ice with polysome buffer (15 mM Tris- Hcl pH 7.4, 1% triton x100, 15 mM MgCl_2_, 0.3M NaCl, 0.1 μg/ml cycloheximide, 1 mg/ml heparin). Then, the cell lysate was centrifuged for 12000 g for 10 minutes at 4°C. Lysate supernatant was carefully isolated and seeded onto 10% to 50% sucrose gradients and centrifuged at 39000 rpm sw40 rotor (HIMAC CP80WX HITACHI) for 160 minutes at 4°C. The sucrose gradient was fractionated with the ISCO gradient fractionation system (ISCO Model 160 Gradient former) connected to a UV detector to monitor absorbance at 254 nm and to record the polysome profile.

Cells were then treated with 2 mM puromycin for 2 hours at 37°C followed by two washes on ice with cold PBS. Cells were lysed in buffer containing: 15 mM Tris- Hcl pH 7.4, 1% Triton x100, 15 mM MgCl_2_, 0.3M NaCl, 1 mg/ml heparin, 2 mM puromycin. The lysate was incubated on ice for 10 min and subunits were then separated at 37°C for 20 min. Gradient separation and fractionation were performed as described for cycloheximide treatment.

### Supplementary data

Supplementary Data are available online: Additional file 3: Table S1 and Additional file 8: Table S2, Additional file 1: Figure S1, Additional file 2: Figure S2, Additional file 4: Figure S3, Additional file 5: Figure S4, Additional file 6: Figure S5, Additional file 7: Figure S6.

## Competing interests

The authors declare no competing interests.

## Authors’ contributions

PS carried out the molecular genetic studies and drafted the manuscript. MAS, CK, FI and JB carried out the immunoassays. JSS participated in the microarray assays. BD conceived the study, and participated in its design and coordination and helped to draft the manuscript. All authors read and approved the final manuscript.

## Supplementary Material

Additional file 1: Figure S1 DZIP1 is located predominantly in the cytoplasm, in a granular pattern. HeLa was transfected with pGFP (A-D) or pYFP (E-H). Nuclei were counterstained with DAPI (blue). (I-L) HEK293 and (M-O) hTERT-RPE1 cells were transfected with pDZIP1-YFP (green). (N-O) Ciliary axonemes were labeled with anti-acetylated tubulin antibodies (red). Arrow = basal bodies of primary cilia. (P-R) HEK293 cells were transfected with pDZIP1-GFP. Fluorescence images of a section that was photobleached in a living cell; the fluorescence intensity of the bleached area was monitored. (P) Cells in phase contrast. (Q) Cells before photobleaching (arrow). (R) Cells two seconds after photobleaching (arrow). (S) Fluorescence recovery graph. An asterisk indicates a cell containing a plasmid. Scale bar: 10 μm. Click here for file

Additional file 2: Figure S2 Real-time imaging of DZIP1-GFP aggregation with granules. (A) HeLa cells were transfected with DZIP1-GFP and cultured for 18 hours at 37°C, and were then shifted to 20°C. Fluorescence images were taken at one-second intervals (the time after the temperature shift is indicated in each panel). (B-C) Indirect immunofluorescence staining was carried out to detect the colocalization of DZIP1 (green) and TIA1 (red) or DCP1 (red) in HeLa cells. Nuclei were counterstained with DAPI (blue). (B) Oxidative stress with 0.5 mM sodium arsenite. (C) Oxidative stress with 2 mM sodium arsenite. Scale bar: 10 μm. Click here for file

Additional file 3: Table S1 mRNA Targets of DZIP1. Ratios of Signal Intensity Were Calculated and Genes that Have Fold Change (IP-DZIP1/IP-Control) Greater or Equal to 2.0 Were Listed. Click here for file

Additional file 4: Figure S3 DZIP1 did not interact robustly with the RNA probe. (A) Western-blot analysis of DZIP1-GFP and GFP levels in protein extracts from cells transfected with the corresponding plasmids. The band detected proximally at 120 kDa corresponds to DZIP1-GFP and the band detected at 27 kDa corresponds to free GFP. (B) Western-blot analysis against MYC tag: eluates (1–4) for affinity purification from cells transfected with a construct encoding DZIP1 fused to a histidine tail and a MYC tag (pSECTAG2). These eluates were used in electrophoretic mobility shift assays (EMSA). (C) We investigated whether DZIP1 interacted directly with these RNAs, by performing EMSA with purified DZIP1 protein and polyr A and C (U and G – not shown) probes. TcRBP40 is an RNA-binding protein used as a positive control. IP, immunoprecipitation. Click here for file

Additional file 5: Figure S4 Expression of *DZIP1* and its mRNAs targets is affected by Hh pathway blockaded. (A) HeLa cells were incubated for several time periods, with various concentrations of cyclopamine. Proliferation was evaluated by BrdU incorporation. (B) We analyzed *GLI1* and *DZIP1* mRNA levels in cells treated with various concentrations of cyclopamine by quantitative RT-PCR. (C-D) No change in the percentage apoptotic cells was observed after treatment of the cells with 300 nM cyclopamine for 24 hours. FACS-based apoptosis analysis showed that cyclopamine caused no significant change in the percentages of live, apoptotic and dead cells with respect to control cells. Dot plots for (C) control and (D) cyclopamine-treated cells. Cells were treated with Alexa Fluor 488 annexin V and propidium iodide (Molecular Probe), and subjected to flow cytometry. (E-F) FACS-based cell cycle analysis demonstrated that cyclopamine treatment did not affect the percentages of cells in the G1, S and G2 phases of the cell cycle. A representative histogram of control cells (E) and cyclopamine-treated cells (F) based on the “Dean-Jett-Fox” model. Click here for file

Additional file 6: Figure S5 *DZIP1* knockdown and overexpression do not affect the accumulation or stability of mRNAs associated with DZIP1-containing complexes but modified quantity of stress granules per cell. (A) Quantitative RT-PCR analysis of *PTCH1, BRD8* and *DZIP1* expression 24, 48 and 72 h after the transfection of cells with 1 nM DZIP1 duplex mix (siDZIP1) or 1 nM Scrambled-negative control duplex (siNC1). (B-C) The percentage of apoptotic cells was similar in *Dzip1*-knockdown cells and control cells. FACS-based apoptosis analysis showed that *DZIP1* knockdown caused no significant change in the percentages of live, apoptotic and dead cells with respect to control cells (siNC1). Dot plot of (B) control and (C) *DZIP1*-knockdown cells. Cells were treated with Alexa Fluor 488 annexin V and propidium iodide (Molecular Probe), and subjected to flow cytometry analysis. (D-E) FACS-based cell cycle analysis demonstrated that *DZIP1* knockdown had no effect on the percentages of cells in the G1, S and G2 phases of the cell cycle. A representative histogram of control cells (D) and *DZIP1*-knockdown cells (E) based on the “Dean-Jett-Fox” model. (F) Greater growth of HeLa cells following *DZIP1* knockdown. Cells were counted at the indicated time points and the mean ± SD values of three independent experiments are shown. (G) Representatives images of fields used to count stress granules. Stress granules were labeled with anti-TIA1 antibody in *DZIP1* knockdown cells and control (siNC1). Click here for file

Additional file 7: Figure S6 Half-life of *BRD8, IFT80, SNX2* and *PTCH* mRNAs in *DZIP1*-knockdown and control cells. HeLa cells transfected with siDZIP1 and siNC1 were treated with Act-D for various times, to block mRNA synthesis. Total RNA extraction, cDNA production, and real-time PCR amplification were performed as described in the text. The values shown are the means and standard deviations (SD) of RNA copy number per μg of total RNA from two independent experiments run in triplicate. *P ≤ 0.05; **P ≤ 0.01. Click here for file

Additional file 8: Table S2 Primer Sets Used for Quantitative Reverse Transcription–Polymerase Chain Reaction Analyses. Click here for file
