# Supplementary figures and images for: Ribonomic analysis of human DZIP1 reveals its involvement in ribonucleoprotein complexes and stress granules
Source: BMC Mol Biol. 2014 Jul 3;15:12. doi: 10.1186/1471-2199-15-12 (PMC4091656; doi:10.1186/1471-2199-15-12)

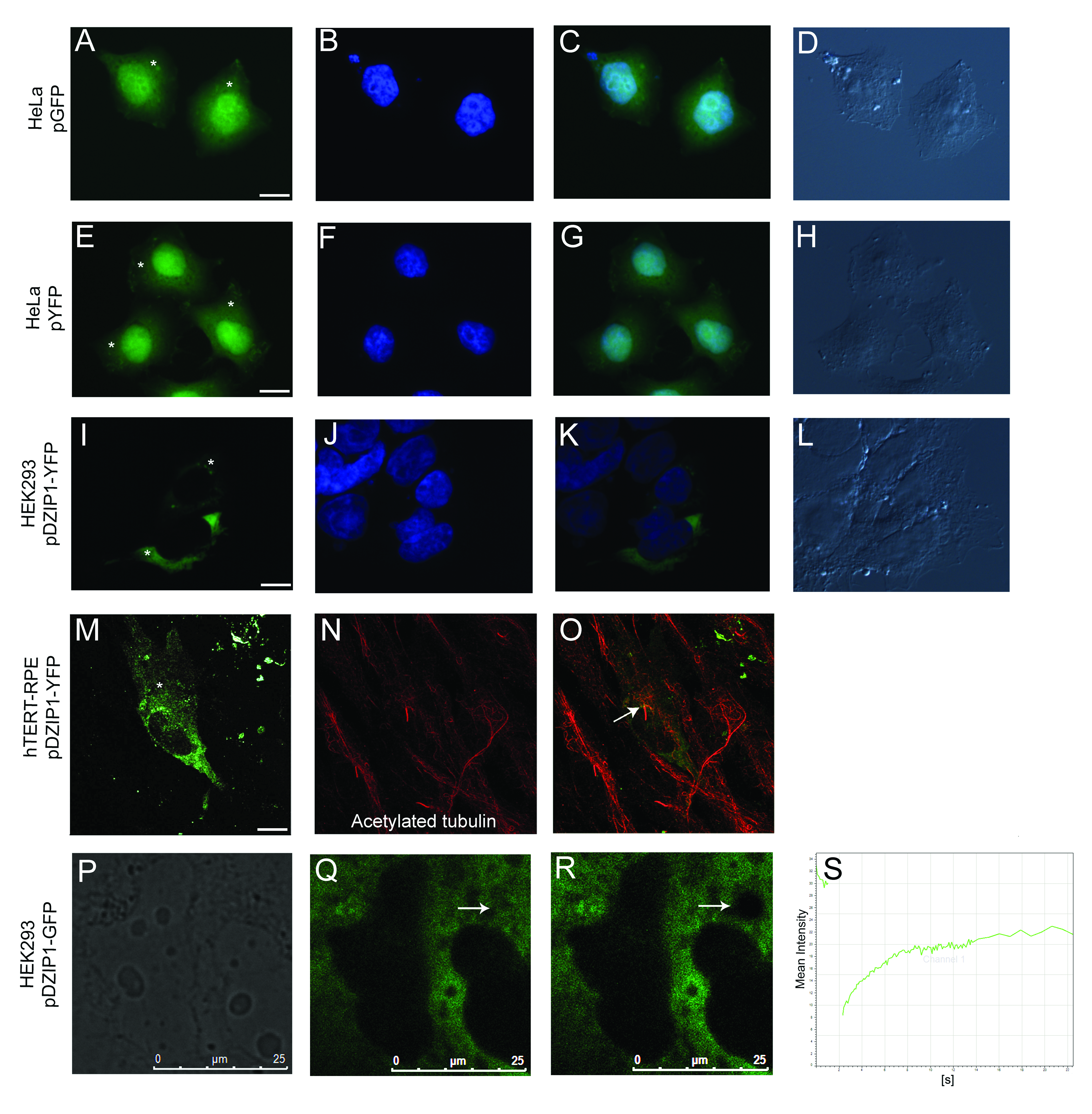

Supplement: Additional file 1: Figure S1 — DZIP1 is located predominantly in the cytoplasm, in a granular pattern. HeLa was transfected with pGFP (A-D) or pYFP (E-H). Nuclei were counterstained with DAPI (blue). (I-L) HEK293 and (M-O) hTERT-RPE1 cells were transfected with pDZIP1-YFP (green). (N-O) Ciliary axonemes were labeled with anti-acetylated tubulin antibodies (red). Arrow = basal bodies of primary cilia. (P-R) HEK293 cells were transfected with pDZIP1-GFP. Fluorescence images of a section that was photobleached in a living cell; the fluorescence intensity of the bleached area was monitored. (P) Cells in phase contrast. (Q) Cells before photobleaching (arrow). (R) Cells two seconds after photobleaching (arrow). (S) Fluorescence recovery graph. An asterisk indicates a cell containing a plasmid. Scale bar: 10 μm. [file 1471-2199-15-12-S1.tiff]

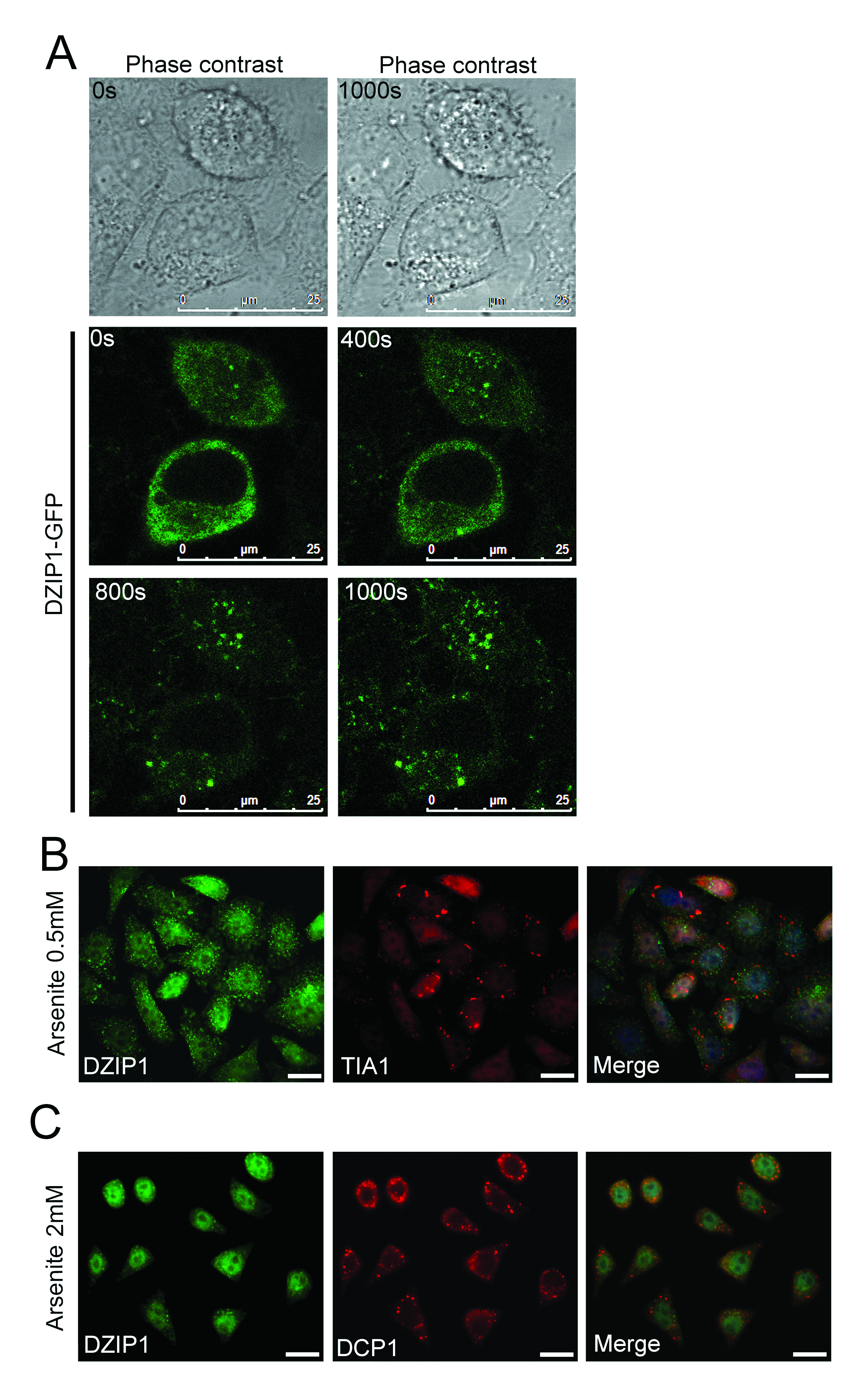

Supplement: Additional file 2: Figure S2 — Real-time imaging of DZIP1-GFP aggregation with granules. (A) HeLa cells were transfected with DZIP1-GFP and cultured for 18 hours at 37°C, and were then shifted to 20°C. Fluorescence images were taken at one-second intervals (the time after the temperature shift is indicated in each panel). (B-C) Indirect immunofluorescence staining was carried out to detect the colocalization of DZIP1 (green) and TIA1 (red) or DCP1 (red) in HeLa cells. Nuclei were counterstained with DAPI (blue). (B) Oxidative stress with 0.5 mM sodium arsenite. (C) Oxidative stress with 2 mM sodium arsenite. Scale bar: 10 μm. [file 1471-2199-15-12-S2.tiff]

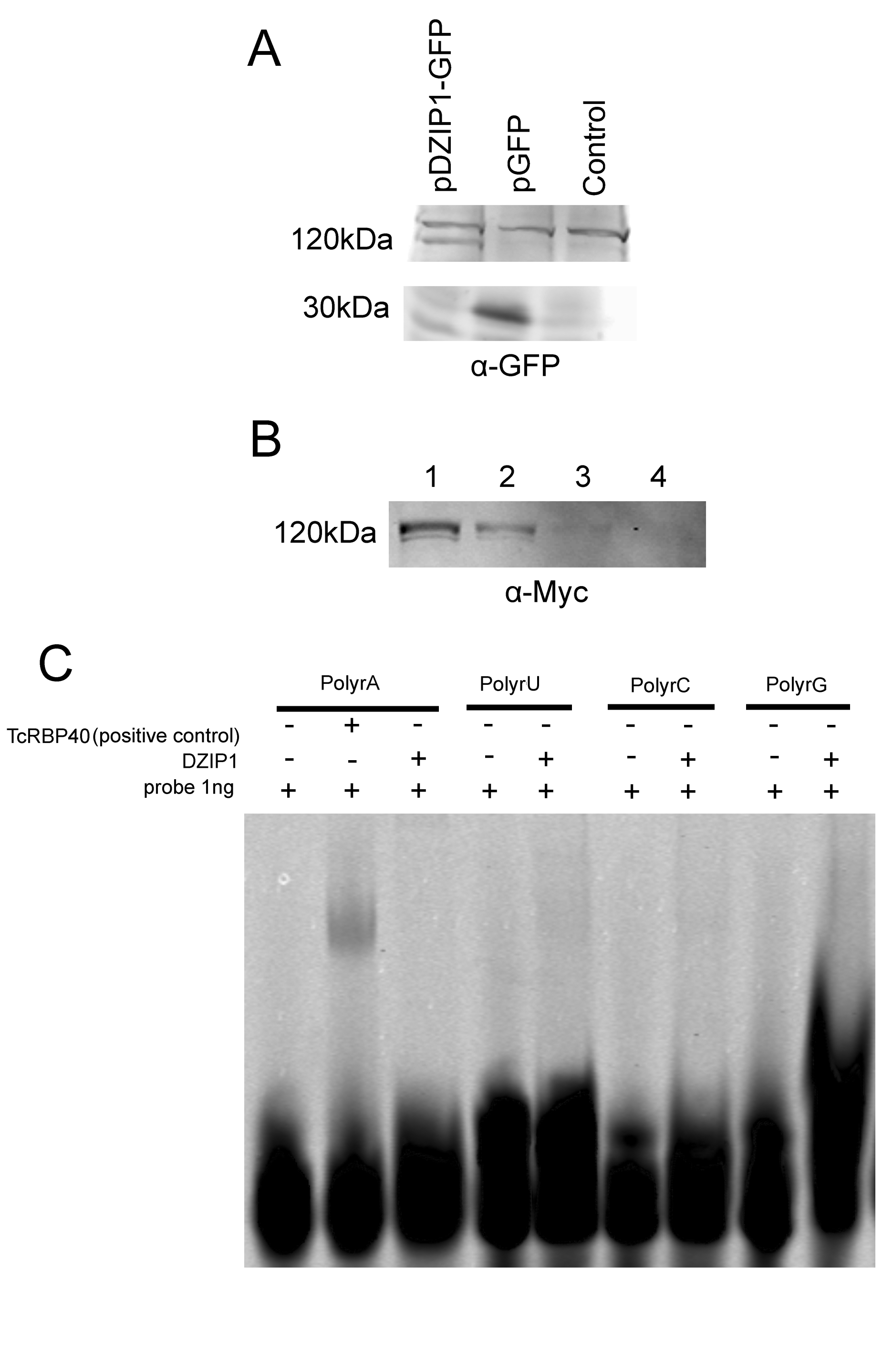

Supplement: Additional file 4: Figure S3 — DZIP1 did not interact robustly with the RNA probe. (A) Western-blot analysis of DZIP1-GFP and GFP levels in protein extracts from cells transfected with the corresponding plasmids. The band detected proximally at 120 kDa corresponds to DZIP1-GFP and the band detected at 27 kDa corresponds to free GFP. (B) Western-blot analysis against MYC tag: eluates (1–4) for affinity purification from cells transfected with a construct encoding DZIP1 fused to a histidine tail and a MYC tag (pSECTAG2). These eluates were used in electrophoretic mobility shift assays (EMSA). (C) We investigated whether DZIP1 interacted directly with these RNAs, by performing EMSA with purified DZIP1 protein and polyr A and C (U and G – not shown) probes. TcRBP40 is an RNA-binding protein used as a positive control. IP, immunoprecipitation. [file 1471-2199-15-12-S4.tiff]

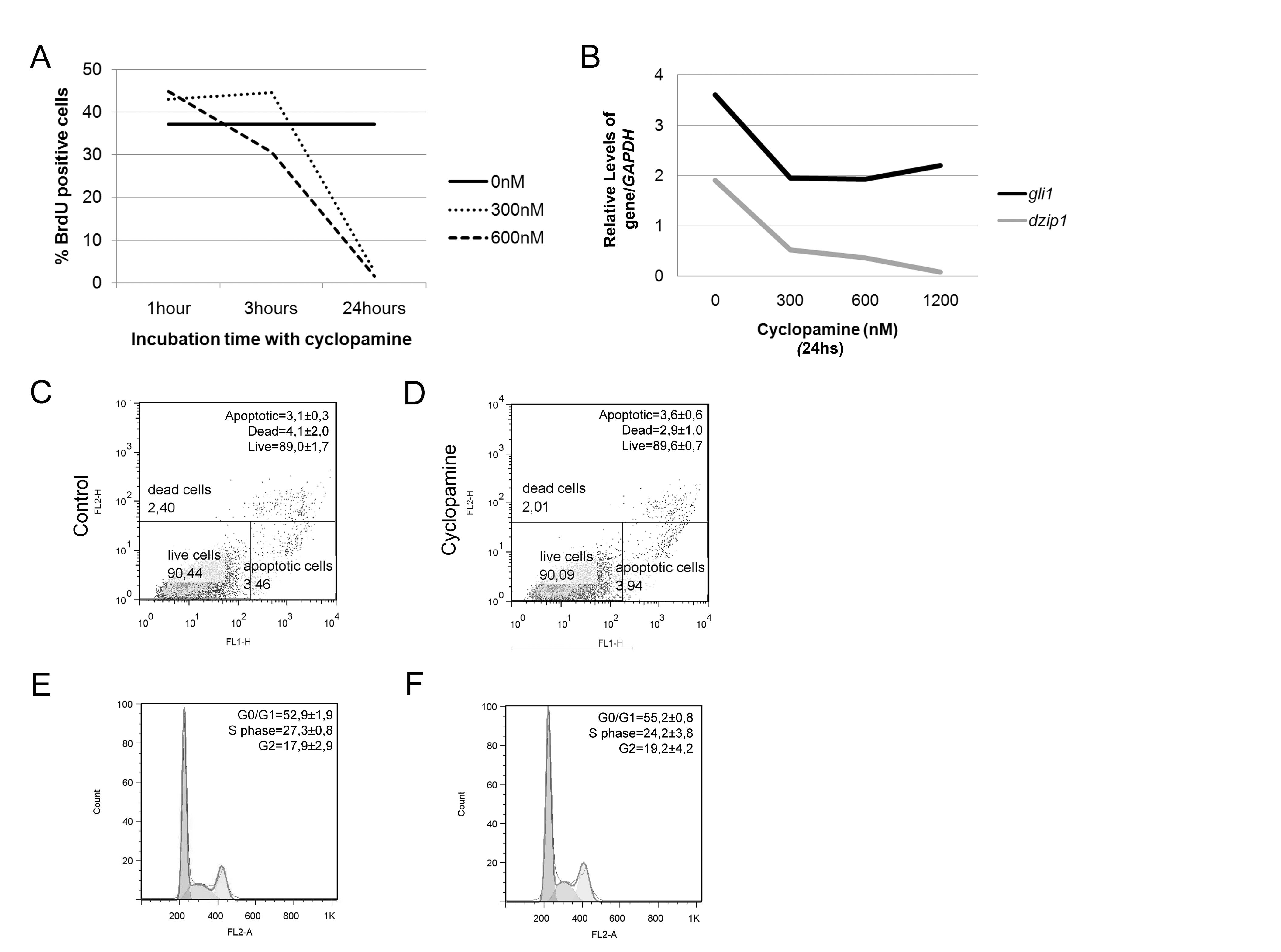

Supplement: Additional file 5: Figure S4 — Expression of DZIP1 and its mRNAs targets is affected by Hh pathway blockaded. (A) HeLa cells were incubated for several time periods, with various concentrations of cyclopamine. Proliferation was evaluated by BrdU incorporation. (B) We analyzed GLI1 and DZIP1 mRNA levels in cells treated with various concentrations of cyclopamine by quantitative RT-PCR. (C-D) No change in the percentage apoptotic cells was observed after treatment of the cells with 300 nM cyclopamine for 24 hours. FACS-based apoptosis analysis showed that cyclopamine caused no significant change in the percentages of live, apoptotic and dead cells with respect to control cells. Dot plots for (C) control and (D) cyclopamine-treated cells. Cells were treated with Alexa Fluor 488 annexin V and propidium iodide (Molecular Probe), and subjected to flow cytometry. (E-F) FACS-based cell cycle analysis demonstrated that cyclopamine treatment did not affect the percentages of cells in the G1, S and G2 phases of the cell cycle. A representative histogram of control cells (E) and cyclopamine-treated cells (F) based on the “Dean-Jett-Fox” model. [file 1471-2199-15-12-S5.tiff]

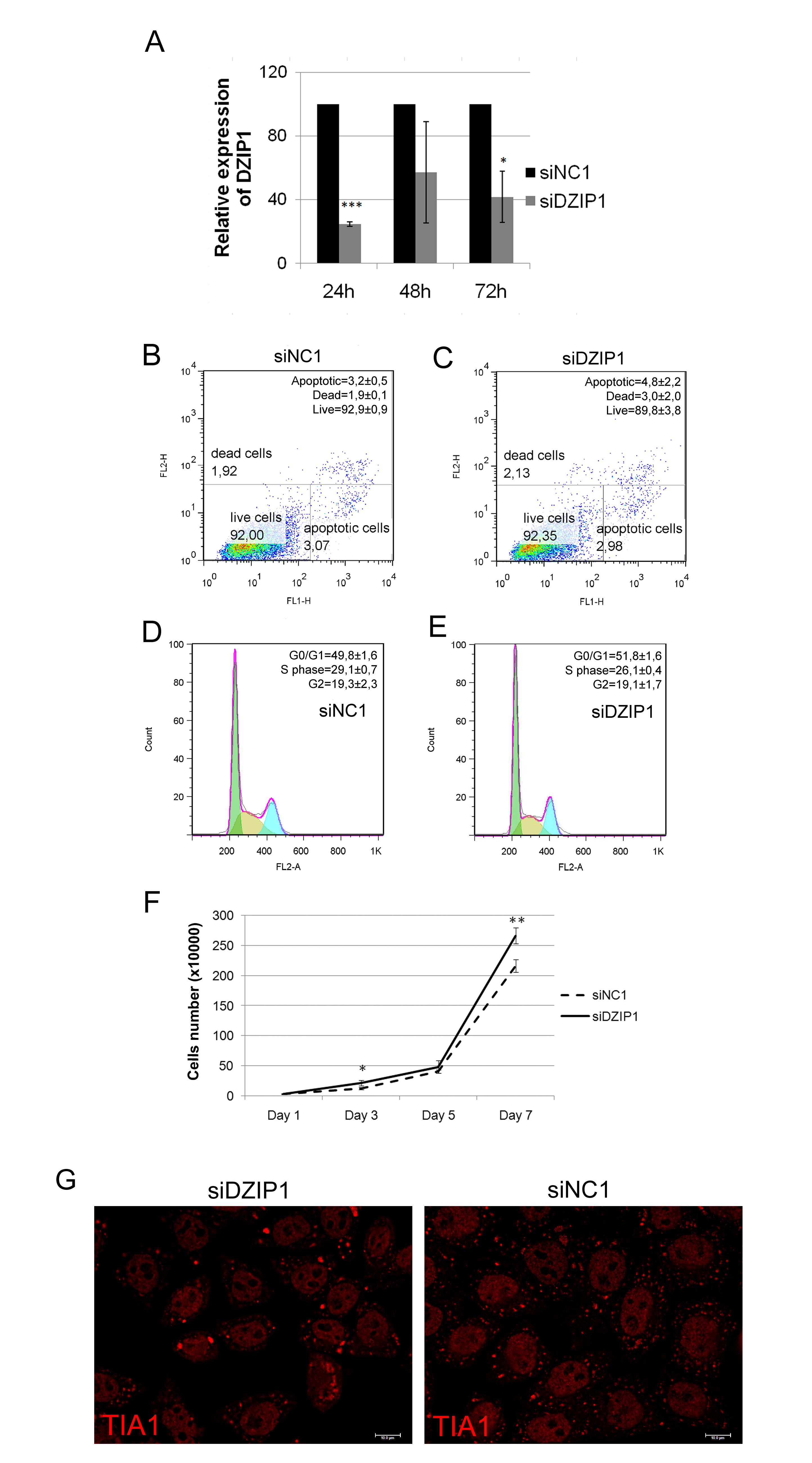

Supplement: Additional file 6: Figure S5 — DZIP1 knockdown and overexpression do not affect the accumulation or stability of mRNAs associated with DZIP1-containing complexes but modified quantity of stress granules per cell. (A) Quantitative RT-PCR analysis of PTCH1, BRD8 and DZIP1 expression 24, 48 and 72 h after the transfection of cells with 1 nM DZIP1 duplex mix (siDZIP1) or 1 nM Scrambled-negative control duplex (siNC1). (B-C) The percentage of apoptotic cells was similar in Dzip1-knockdown cells and control cells. FACS-based apoptosis analysis showed that DZIP1 knockdown caused no significant change in the percentages of live, apoptotic and dead cells with respect to control cells (siNC1). Dot plot of (B) control and (C) DZIP1-knockdown cells. Cells were treated with Alexa Fluor 488 annexin V and propidium iodide (Molecular Probe), and subjected to flow cytometry analysis. (D-E) FACS-based cell cycle analysis demonstrated that DZIP1 knockdown had no effect on the percentages of cells in the G1, S and G2 phases of the cell cycle. A representative histogram of control cells (D) and DZIP1-knockdown cells (E) based on the “Dean-Jett-Fox” model. (F) Greater growth of HeLa cells following DZIP1 knockdown. Cells were counted at the indicated time points and the mean ± SD values of three independent experiments are shown. (G) Representatives images of fields used to count stress granules. Stress granules were labeled with anti-TIA1 antibody in DZIP1 knockdown cells and control (siNC1). [file 1471-2199-15-12-S6.tiff]

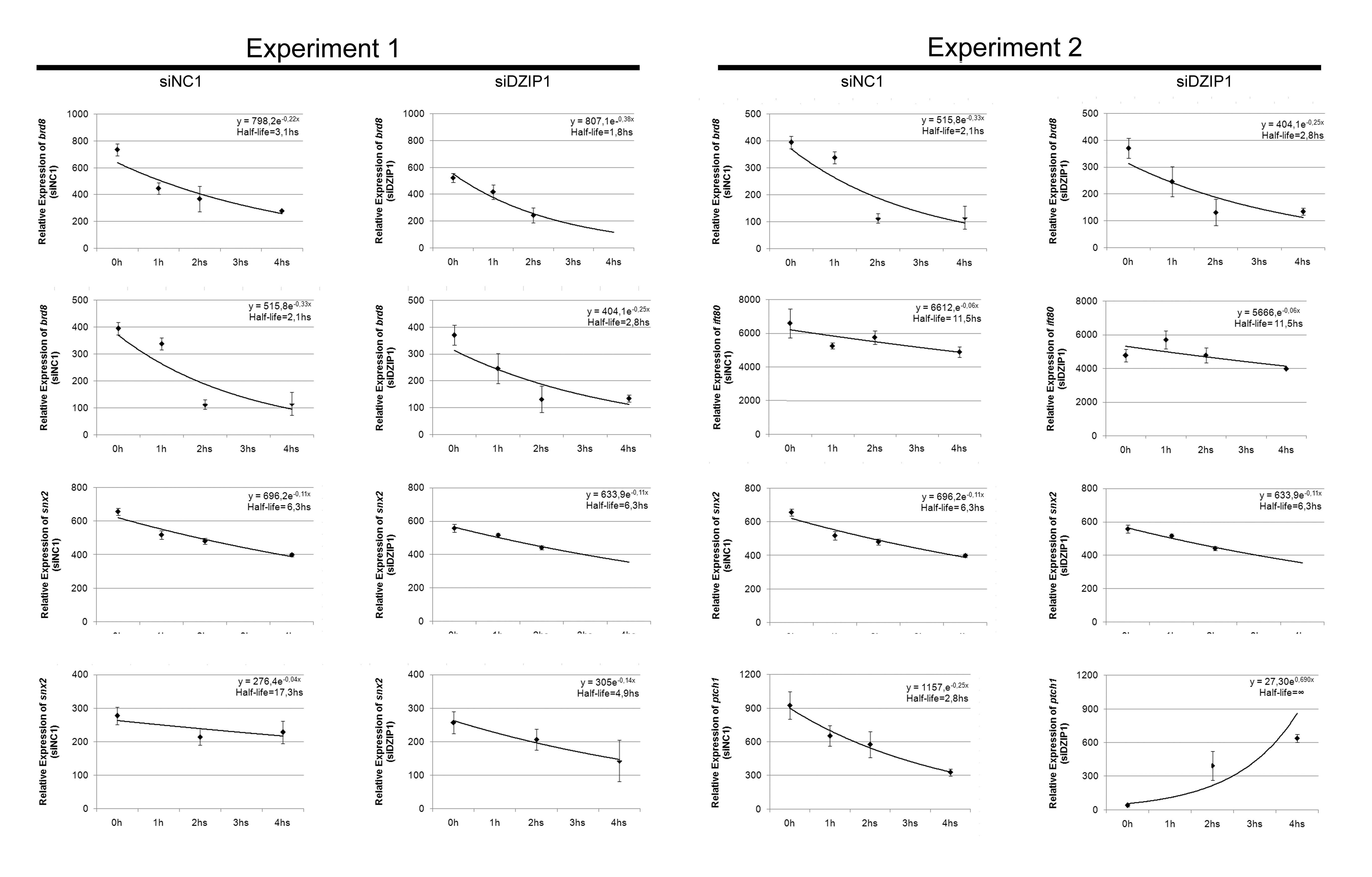

Supplement: Additional file 7: Figure S6 — Half-life of BRD8, IFT80, SNX2 and PTCH mRNAs in DZIP1-knockdown and control cells. HeLa cells transfected with siDZIP1 and siNC1 were treated with Act-D for various times, to block mRNA synthesis. Total RNA extraction, cDNA production, and real-time PCR amplification were performed as described in the text. The values shown are the means and standard deviations (SD) of RNA copy number per μg of total RNA from two independent experiments run in triplicate. *P ≤ 0.05; **P ≤ 0.01. [file 1471-2199-15-12-S7.tiff]
