# Supplementary material for: Ribonomic analysis of human DZIP1 reveals its involvement in ribonucleoprotein complexes and stress granules
Source: BMC Mol Biol. 2014 Jul 3;15:12. doi: 10.1186/1471-2199-15-12 (PMC4091656; doi:10.1186/1471-2199-15-12)
Supplement: Additional file 8: Table S2 — Primer Sets Used for Quantitative Reverse Transcription–Polymerase Chain Reaction Analyses. [file 1471-2199-15-12-S8.doc]

| **Supplementary Table S2.** Primer Sets Used for Quantitative Reverse Transcription–Polymerase Chain Reaction Analyses | | | |
| --- | --- | --- | --- |
| **Official symbol** | **NCBI ID** | **Primer sequence (5´–3´)** | **Amplicon (bp)** |
| DZIP1 | NM_014934 | Forward GCCATCGACGTGGACAAGGTGGC | 205 |
|  |  | Reverse TGTGACTTTGTAGAAAAGCTTGG |  |
| GAPDH | NM_002046.3 | Forward: GGCGATGCTGGCGCTGAGTAC | 149 |
|  |  | Reverse: TGGTTCACACCCATGACGA |  |
| SNX2 | NM_003100.2 | Forward: GACGGAGAGGACCTGTTCAC | 240 |
|  |  | Reverse CAGGTGTGACTGCAGGAGAA |  |
| PTCH1 | NM_001083606.1 | Forward: ATCCATGTGGCTGCCCTCTT | 223 |
|  |  | Reverse CACAGCTCCTCCACGTTGGT |  |
| IFT80 | NM_020800.1 | Forward: GGGATGCTTAGATCAACTTTAGCTC | 159 |
|  |  | Reverse: GCCATCATGAGCTTTCCAC |  |
| BRD8 | NM_006696.3 | Forward: GCGACGGGAACGGGCAAACA | 157 |
|  |  | Reverse: TCTGGAGGGCGGCCAGGTTC |  |
| GLI1 | NM_005269.2 | Forward: CCCGCCCTTCTGCCACCAAG | 182 |
|  |  | Reverse: ACCGTCTGCAGGTCCAGGCT |  |
| PUM1 | NM_001020658.1 | Forward: AAACCTGAGAAGTTTGAATTG | 351 |
|  |  | Reverse: GCAAGACCAAAAGCAGAGTTG |  |
| DISP1 | NM_032890.2 | Forward: GAGCTGCGCCTGCCAACTCA | 231 |
|  |  | Reverse: CAGGGGGTGAGGGGACTCGG |  |
| NPC1 | NM_000271.4 | Forward: ACGCCTTCTTCCTTCCTGA | 292 |
|  |  | Reverse: ACCTCTTGTCCCCATATGCAA |  |
| CSNK1E | NM_001894.4 | Forward: TCCTCTCTGTGCCCATCACA | 162 |
|  |  | Reverse: ATATCTCCGAAGGACCCGCT |  |
| MYO5B | NM_001080467.2 | Forward: CGGCGGGAGTAAAGGTCG | 266 |
|  |  | Reverse: CGCCATACCTCATCAGGGTC |  |
| CEP164 | NM_001271933.1 | Forward: ACAGGTGTTTGAGCCCAGAT | 259 |
|  |  | Reverse: TTCGTCACATGGATGGTCCC |  |
| STK36 | NM_001243313.1 | Forward: CCACCCCAGATTGTGAACGA | 140 |
|  |  | Reverse: AGGCTCAGTGGTCTCTAGCA |  |
| ASH1L | NM_018489.2 | Forward: TAGCCTTCCACCTTTCCCTTC | 297 |
|  |  | Reverse: TCTAGGGTCCATCACAAGCG |  |
| DZIP1, DAZ interacting protein 1; GAPDH, glyceraldehyde-3-phosphate dehydrogenase; SNX2, sorting nexin 2; PTCH1, patched homolog 1 (Drosophila); IFT80, intraflagellar transport 80 homolog; BRD8, bromodomain containing 8; GLI1, GLI family zinc finger 1; PUM1, pumilio homolog 1; DISP1, dispatched homolog 1; NPC1, Niemann-Pick disease, type C1; CSNK1E, casein kinase 1, épsilon; MYO5B, myosin VB; CEP164, centrosomal protein 164kDa; STK36, serine/threonine kinase 36; ASH1L, (absent, small, or homeotic)-like (Drosophila). | | | |
